# Low-Dimensional Models of “Neuro-Glio-Vascular Unit” for Describing Neural Dynamics under Normal and Energy-Starved Conditions

**DOI:** 10.3389/fneur.2016.00024

**Published:** 2016-03-09

**Authors:** Karishma Chhabria, V. Srinivasa Chakravarthy

**Affiliations:** ^1^Computational Biophysics and Neurosciences Laboratory, Department of Biotechnology, Bhupat and Jyoti Mehta School of Biosciences, Indian Institute of Technology Madras, Chennai, India

**Keywords:** neuro-glio-vascular, ATP, firing threshold, bursting, LFP

## Abstract

The motivation of developing simple minimal models for neuro-glio-vascular (NGV) system arises from a recent modeling study elucidating the bidirectional information flow within the NGV system having 89 dynamic equations (1). While this was one of the first attempts at formulating a comprehensive model for neuro-glio-vascular system, it poses severe restrictions in scaling up to network levels. On the contrary, low-­dimensional models are convenient devices in simulating large networks that also provide an intuitive understanding of the complex interactions occurring within the NGV system. The key idea underlying the proposed models is to describe the glio-vascular system as a lumped system, which takes neural firing rate as input and returns an “energy” variable (analogous to ATP) as output. To this end, we present two models: biophysical neuro-energy (Model 1 with five variables), comprising K_ATP_ channel activity governed by neuronal ATP dynamics, and the dynamic threshold (Model 2 with three variables), depicting the dependence of neural firing threshold on the ATP dynamics. Both the models show different firing regimes, such as continuous spiking, phasic, and tonic bursting depending on the ATP production coefficient, ɛ_p_, and external current. We then demonstrate that in a network comprising such energy-dependent neuron units, ɛ_p_ could modulate the local field potential (LFP) frequency and amplitude. Interestingly, low-frequency LFP dominates under low ɛ_p_ conditions, which is thought to be reminiscent of seizure-like activity observed in epilepsy. The proposed “neuron-energy” unit may be implemented in building models of NGV networks to simulate data obtained from multimodal neuroimaging systems, such as functional near infrared spectroscopy coupled to electroencephalogram and functional magnetic resonance imaging coupled to electroencephalogram. Such models could also provide a theoretical basis for devising optimal neurorehabilitation strategies, such as non-invasive brain stimulation for stroke patients.

## 1 Introduction

A key tenet of the contemporary neuroscience states that neurons constitute the primary units of brain’s information processing networks. However, there is growing evidence suggesting an imperative role of the “other brain” in sustaining the brain’s physiological activity (2–4). This other brain comprises the glial cells that occupy around half of the brain’s volume, though the exact numbers and neuron/glia ratio vary across the brain (5–8). Developments in glial research over the last two decades reveal the immense and extensive contributions of this system to brain functions, such as neurotransmitter homeostasis, potassium siphoning, and shuttling the energy substrates across the blood–brain barrier among others (2, 9–18). Interestingly, glial cells also sense and modulate the synaptic activity (19, 20) in addition to the above-mentioned maintenance functions. There are significant studies speculating on the contributions of glial cells in brain’s computations (21, 22).

Neural activity is constantly sensed by a type of glial cells called the astrocytes, whose perisynaptic processes eavesdrop on ongoing neurotransmission events (23–25). The end-feet of astrocytes also wrap around the blood vessels, thereby forming the blood–brain barrier (26). This configuration is known to facilitate the transmission of “hunger signals” from the neurons to the cerebral blood vessels through the glial interface (27, 28). The possibility of reverse influence from the vessels to the neurons is generally neglected, though there are experimental grounds supporting the role of vasomotion in various diseases, such as diabetes, hypertension, and even Alzheimer’s disease (29, 30). Recent studies present substantial evidence supporting the role of glio-vascular dysfunction in cognitive impairments, such as epilepsy, neurodegenerative disorders, and migraine (31–33). Furthermore, some recent proposals postulate a role for the glio-vascular system in neural information processing (1, 34–36).

These significant developments in glial and cerebrovascular research indicate a need to incorporate both the glial and vascular systems in an expanded theory of brain’s computations. Hence, it seems pragmatic to investigate further the role of glial cells and the cerebral vasculature in information processing in the brain. Therefore, we hypothesize that the neural activity also has an obligate dependence on the spatiotemporal vascular dynamics governed by the astrocyte activity.

Chander and Chakravarthy (1) proposed a model of the neuro-glio-vascular (NGV) system, in which a single neuron interacts with a single astrocyte and single microvessel. The model is a detailed biophysical model consisting of 89 dynamic equations. In order to explore, using computational models, the possible role of NGV system as a fundamental unit in brain’s information processing, it is essential to develop network models of the NGV system. However, with a model that is significantly complex at single-unit level, it is difficult to scale up to the network level. Therefore, the main objective of the present study was to formulate simple models of the NGV system whose rationale is inspired by the behaviors observed in more complex models like that of Chander and Chakravarthy (1). Considering the serious challenges involved in systematically reducing an 89-dimensional system to a five-variable system, we begin with a simple five-variable biophysical neuron model that captures the dependence of neural firing on ATP. This five-variable biophysical model is constructed by modifying the neuron model of Ching et al. (37).

**Figure 1:**
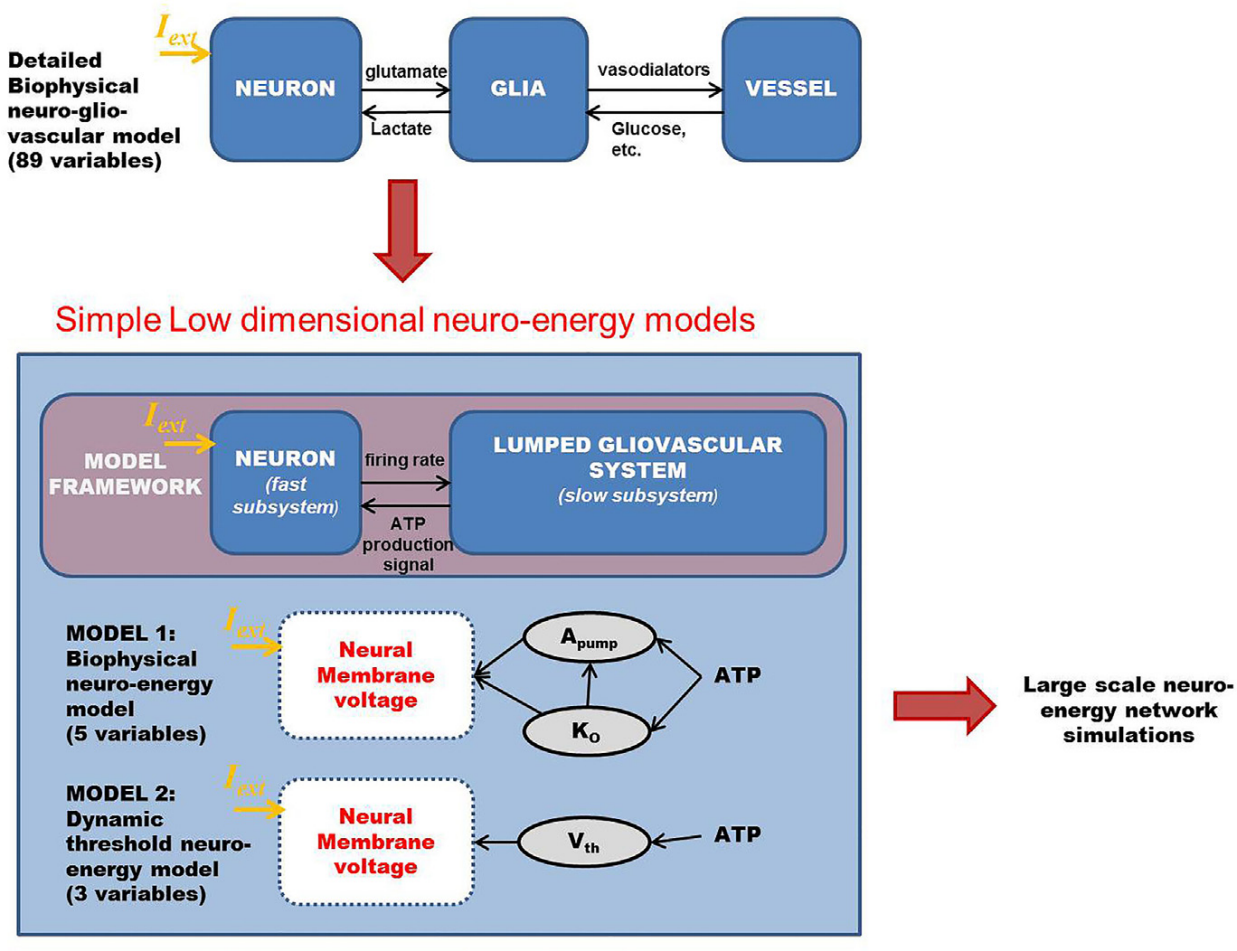
**Schematic representation of the proposed modeling framework for the low-dimensional models of “neurovascular unit,” wherein the glio-vascular system is lumped into a single module**. Model 1 is biophysical with neuronal ATP affecting the Na^+^–K^+^ ATPase pump activity (*A*_pump_) and extracellular potassium ion concentrations (K_o_), which eventually governs the neuronal membrane potential. Model 2 is an abstract model with neuronal ATP modulating the firing threshold (*V*_th_) of a quadratic integrate-and-fire model. *I*_ext_ is the common key parameter across all the models and represents the external input current to the neuron (in microampere per square centimeter).

In an attempt to develop a more generic, low-dimensional model that shows the effects of varying energy (ATP) levels in a spiking neuron model as a function of vessel dynamics, we have developed two models (Figure 1). Our approach to development of the proposed simplified model of the NGV system is as follows: instead of treating the astrocyte and the vessels as independent, isolated entities, we represent the entire glial-vascular system as a single, lumped system, which represents a source of energy substrates for the neurons. Thus, the proposed system has two modules: a neuron module and an “energy” module. The output of the neuron module is its firing activity, which is sensed by the energy module. In turn, the energy module supplies “energy” to the neuron module to fuel its firing activity. Since the neuron module is characterized by the fast neural dynamics, it is considered the fast subsystem. The “energy” module, which represents the slower glial and vascular dynamics, is the slow subsystem. The energy module takes the firing activity output of the neuron module and releases energy in the form of ATP. It must be noted that the firing activity of the neuron has a dual impact on the ATP dynamics: on the one hand, neural firing activity leads to the consumption of ATP *via* the activity of Na^+^–K^+^ ATPase pump, while on the other hand, it acts as a trigger to induce the energy module to release more energy in the form of ATP (38). Based on this paradigm, we first present the two minimal models for NGV. The first minimal model (Model 1 with five dynamic variables) described in the present study is biophysical and elucidates the effect of intracellular [ATP] on the excitability of a mammalian cortical pyramidal neuron by modulating the K_ATP_ channel activity. The first model reproduces most of the dynamical behaviors (such as tonic spiking and tonic bursting) of the detailed model. We then propose that this regulatory effect of [ATP] changes the neuronal *firing threshold* and thereby governs its excitability. Accordingly, we propose the second model (Model 2 with three dynamic variables), which comprises a quadratic integrate-and-fire neuron with a dynamic threshold, governed by intracellular [ATP].

In both the models, ATP consumption directly depends on neural spiking activity. The production rate coefficient of ATP, ɛ_p_, is a crucial parameter that is proposed to represent local vascular activity. Furthermore, the simpler Model 2 is calibrated against the biophysical Model 1 so as to obtain similar spiking rates. The neural dynamics in both the models expresses the same behaviors (tonic spiking, phasic bursting, and tonic bursting) for a similar range of control parameters: production rate coefficient of ATP, ɛ_p_, and external input current, *I*_ext_.

We then describe a network model comprising one of the described “neuro-energy” models, followed by analyzing the model behavior under physiological and energy-starved conditions. This is done by calculating local field potentials (LFPs) and comparing the frequency spectrum for different values of control parameters.

## 2 Materials and Methods

The simple models proposed in this study are canonical and are formulated to study further the relation between firing patterns and intraneuronal ATP, as was observed in the detailed biophysical model (1). We begin with a five-variable biophysical model that instantiates the dependence of neural firing on ATP and closely relates to the model of Ching et al. (37). Model behavior is studied by varying two parameters: ɛ_p_ and *I*_ext_. The first parameter ɛ_p_ controls ATP production rate. Reduced values of parameter, ɛ_p_, leads to slow-down in ATP production as, for example, in the case of a constricted vessel. The second key parameter of both the models is *I*_ext_, which represents the input current received by the neuron. The neurons activity is also critically governed by *I*_ext_ such that below a threshold value of the current, the neuron does not spike at all as is the conventional approach to modeling biophysical neuron. In the models described in the following sections, we demonstrate the physiological range of these parameters, wherein the neuron shows continuous firing and the pathological range (especially ɛ_p_), wherein the neuron enters burst firing modes.

### 2.1 Model 1: Biophysical Neuro-Energy Model

**Figure 2:**
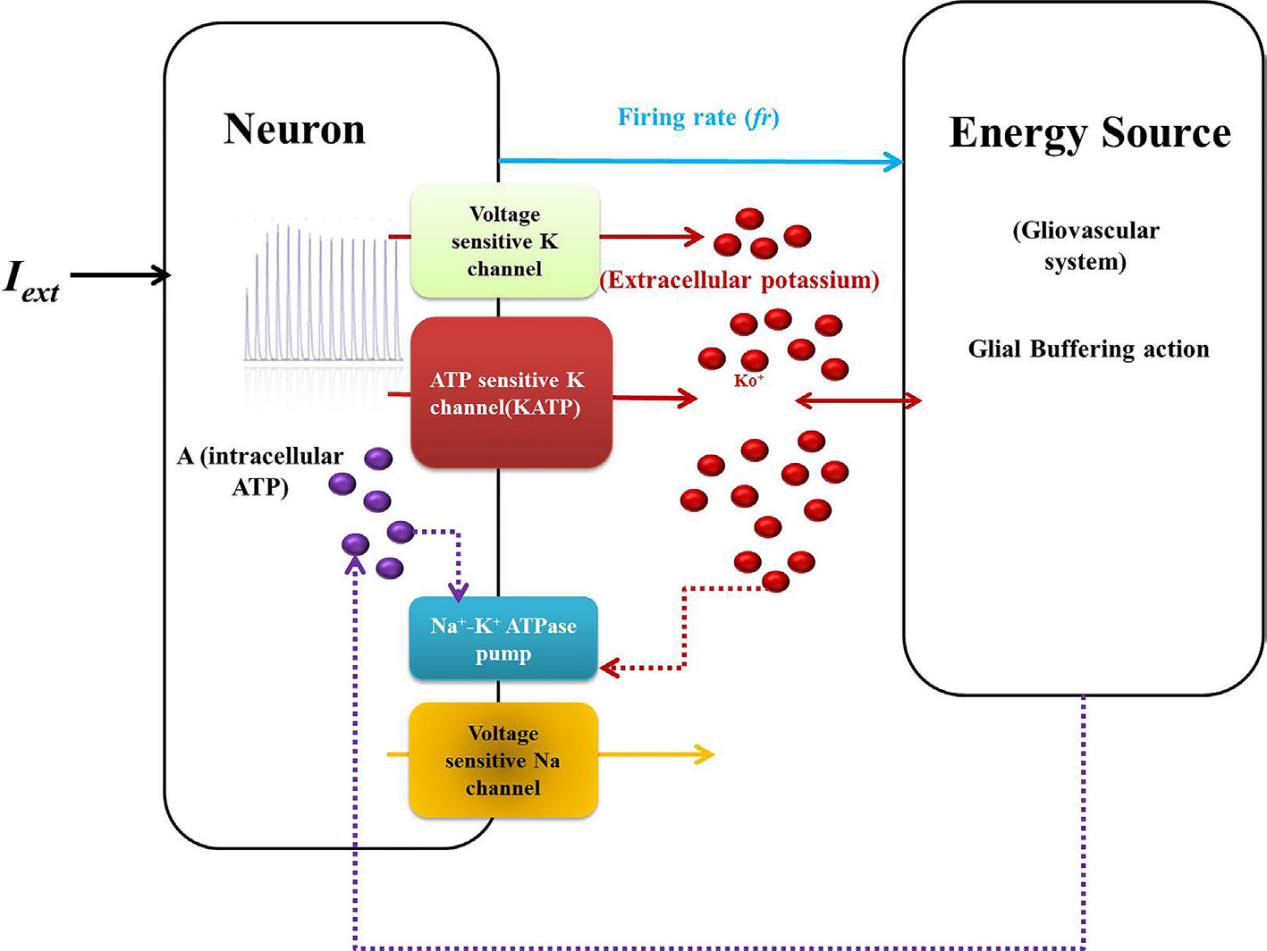
**Schematic representation of Model 1: biophysical model: the neuron comprises the first compartment with the corresponding ion channels**. The second compartment represents the glio-vascular system influencing the activity of the first *via* two mechanisms: (a) buffering of the K_o_ and (b) regulation of production rate of ATP (ɛ_p_). The input to the glio-vascular system is the delayed firing rate signal from the neuron.

Model 1 is described by five dynamic variables with the neuron ­represented by two variables: membrane voltage, *V*, and Na^+^ channel gating variable, *n* [Eq. (1)]. The typical Hodgkin–Huxley-based mammalian cortical neuron model is reduced to two variables by assuming the gating variables *m* to be extremely fast and *h* to be slow. Such assumptions are regularly made to construct low-dimensional neuron models, for example, the FitzHugh–Nagumo model (39, 40). This makes *m* simply a function of voltage, *V* (Eq. 2) and *h*, a constant (Eq. 4). An important characteristic of the model is the additional K_ATP_ channel (41), which is an ATP-dependent potassium channel and forms a direct link between metabolism and neuronal activity. The schematic representation of the model is given in Figure 2.

(1) $$\begin{array}{ll} C\frac{\text{d}V}{\text{dt}} & +g_{\text{Na}}m^{3}h(V-E_{\text{Na}})+g_{\text{K}}n^{4}(V-E_{\text{K}})\\ & +g_{K_{\text{ATP}}}z(V-E_{\text{K}})+g_{\text{L}}(V-E_{\text{L}})=I_{\text{ext}} \end{array}$$

where

(2) $$m(V)=\frac{\alpha_{\text{m}}(V)}{\alpha_{\text{m}}(V)+\beta_{\text{m}}(V)}$$

and

(3) $$\frac{\text{d}n}{\text{dt}}=\alpha_{\text{n}}(V)(1-n)-\beta_{\text{n}}(V)n$$

(4) h=0.1

(5) $$\alpha_{\text{m}}=\frac{0.32(V+54)}{1-e^{-\frac{V+54}{4}}}$$

(6) $$\beta_{m}=\frac{0.28(V+27)}{e^{\frac{V+27}{5}-1}}$$

(7) $$\alpha_{\text{n}}=\frac{0.32(V+52)}{1-e^{-\frac{V+52}{5}}}$$

(8) $$\beta_{\text{n}}=0.5e^{-\frac{V+57}{40}}$$

The corresponding forward and backward rate functions, α_m_(*V*), β_m_(*V*), α_n_(*V*), and β_m_(*V*), given by Eqs 5–8 (37). In addition, *C* is the membrane capacitance, *g*_i_ and *E*_i_ are the maximal channel conductance and reversal potential, respectively, for the *i*th ion, and $g_{{\text{K}}_{\text{ATP}}}$ is the maximal channel conductance for the K_ATP_ channel gated by variable *z* given by Eq. 9, as implemented by Ching et al. (37).

(9) $$z=\frac{1}{1+10A}$$

where *A* is intracellular [ATP] and *I*_ext_ is the continuous external current. The inverse relation of the gating variable, *z*, with the ATP, *A*, signifies the functionality of the K_ATP_ channels, depicting that the channels would be open under low ATP concentrations and closed at physiological ATP concentrations (37). The values and units of the variables are shown in Table 1.

**Table 1:** **Table for values of different parameters and constants used in the formulation of Model 1**.

Parameters and constants	Values
*E*_Na_	50 mV (37)
*E*_K_	−67 mV (37)
*^g^*Na	100 mS/cm^2^ (37)
*^g^*_K_	80 mS/cm^2^ (37)
$g_{{\text{K}}_{\text{ATP}}}$	0.15 mS/cm^2^ (37)
*^g^*_L_	0.1 mS/cm^2^ (37)
*I*_max_	1.3 μA/cm^2^ (43)
*C*	1 μF/cm^2^ (37)
a	0.066 (current model parameter)
*b*	0.033 (current model parameter)
*c*	0 (current model parameter)
λ_A_	0.009 (current model parameter)
λ_fr_	0.14 (current model parameter)

*The current model parameters *a*, *b*, and *c* are tuned so as to get optimum buffering effect making K_o_ lie within the specific physiological range of 3–10 mM (42)*.

In addition, the K_o_ dynamics affect the membrane voltage through *E*_K_ (in millivolts), which is given by the Nernst potential equation (Eq. 10).

(10) $$E_{\text{K}}=26.7\text{log}(\frac{K_{o}}{{\text{K}}_{\text{tot}}-K_{o}})$$

where

(11) $${\text{K}}_{\text{tot}}=K_{o}+K_{i}$$

where K_i_ and K_o_ are the intracellular and extracellular potassium ion concentrations, respectively. K_tot_ denotes the sum of intracellular and extracellular potassium ion concentrations, fixed at 133 mM for our simulations, assuming the intra- and extra-compartmental volumes to be the same (Eq. 12).

(12) $${\text{Vol}}_{\text{extracellular}}={\text{Vol}}_{\text{intracellular}}$$

The K_o_ dynamics is given by summation of both inward and outward K^+^ currents along with a quadratic term: $f_{\text{glia}}(K_{o})$ (Eq. 14), which denotes the extracellular potassium buffering activity of the glial cells around the neuron. The quadratic term is more appropriate compared to a linear term because at resting conditions, with a linear buffering term, K_o_ can reach 0 value in the absence of buffering, i.e., $f_{\text{glia}}(K_{o})=0$ (which does not happen physiologically). On the contrary, the quadratic term ensures that K_o_ is always non-zero and maintained at the basal physiological value. The constants and other parameters are given in Table 1. Buffering is important as it does not allow K_o_ to go beyond a critical value. Studies have shown that K_o_ needs to be effectively buffered by spatial diffusion and/or glial activity (18). The buffering effect is due to the activity of various channels present on the membrane of glial cells, such as inward-rectifying potassium channels (Kir) and potassium pumps (Na–K and NKCC) (44–46). The activity/conductance of the channels and the pumps depend on the concentration of extracellular potassium (K_o_). The conductance of a Kir channel is described by *g* = *f*(K_o_)*^*n*^*, where *n* varies with the type of cell/neuron (47, 48) and the pump activity depends non-linearly on [K_o_]. However, the combined buffering effect of the channels and the pumps, together, is not explicitly modeled. There are models for potassium buffering, though more detailed than ours, like that of Kager et al. (43), which assume the buffering capacity to be limited. On the other hand, we modeled the buffering term with no constraint on the upper limit of the buffering capacity in order to study the effect of “energy” on the neural excitability, in isolation. Thus, as a simple case, we assume the glial buffering term to be quadratic and encompassing all the effects resulting in decrease in extracellular potassium. The advantage of a quadratic buffering term is that it gives a stable fixed point at a finite value of [K_o_], representing the stable value of [K_o_] sustained by the buffering process. Further experiments are required to validate the buffering term, $f_{\text{glia}}(K_{o})$.

In general, buffering is important as in the absence of buffering, K_o_ accumulates, making the neuron hyperexcitable, a phenomenon observed in cases of epilepsy and spreading depression (49). Few experimental studies also attribute anomalies in Ca^2+^ activated K^+^ channels to the excessive accumulation of K_o_ (50). Thus, we speculate that the non-linear buffering term, $f_{\text{glia}}(K_{o})$, is generic and is representative of the multiple factors affecting the concentration of K_o_.

(13) $$\tau_{\text{K}}\frac{{\text{dK}}_{\text{o}}}{\text{dt}}=-f_{\text{glia}}(K_{o})+\pi_{\text{S}}I_{{\text{K}}_{\text{tot}}}$$

where

(14) $$f_{\text{glia}}(K_{o})=aK_{o}^{2}+bK_{o}+c$$

and

(15) $$I_{{\text{K}}_{\text{tot}}}=I_{K_{o}}+I_{K_{i}}+I_{{\text{K}}_{\text{ATP}}}$$

where *a*, *b*, and *c* are buffering constants and π_S_ is the “current density to concentration” conversion factor (1), whose values are given in Table 1. $I_{K_{o}}$ is outward potassium current (Eq. 16), $I_{K_{i}}$ is the inward potassium pump current (Eq. 17), and $I_{{\text{K}}_{\text{ATP}}}$ is the outward potassium current through the ATP-dependent potassium channel (Eq. 18). The various ion channel current equations are described by Eqs 16–18.

(16) $$I_{K_{o}}=g_{\text{K}}n^{4}(V-E_{\text{K}})$$

(17) $$I_{K_{i}}=-2I_{\text{max}}A_{\text{pump}}$$

and

(18) $$I_{{\text{K}}_{\text{ATP}}}=g_{{\text{K}}_{\text{ATP}}}z(V-E_{\text{K}})$$

**Figure 3:**
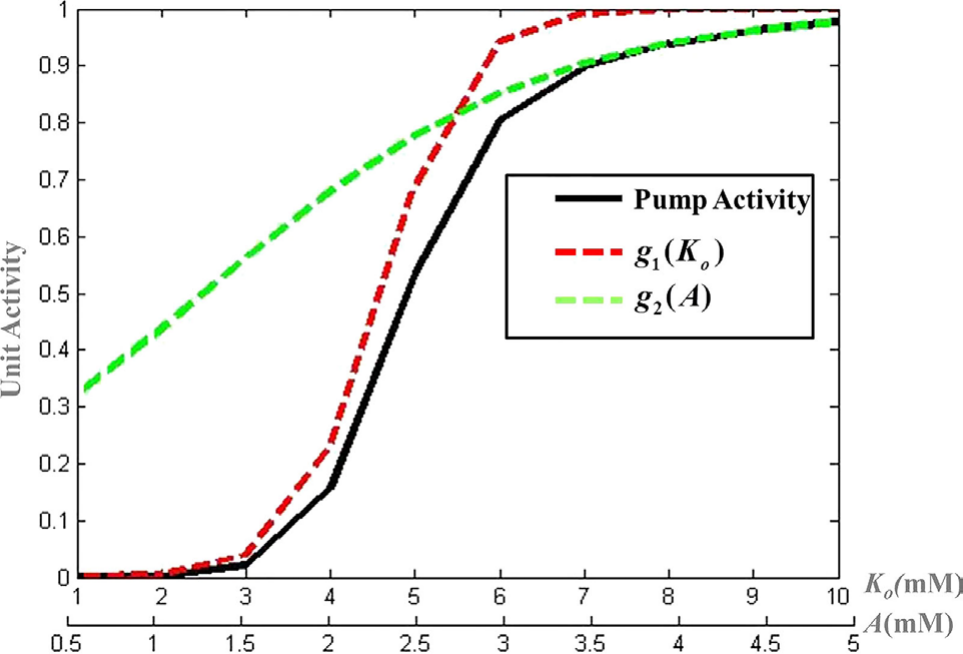
**Modeled activity of Na^+^–K^+^ ATPase pump, where $g_{1}\left(K_{o}\right)=1+{\text{e}}^{-\text{λ}\left(K_{o}-\text{k}\right)}$ and *g*_2_(*A*) = 1 + *e*^−μ(^*^A^*^ − ^*^l^*^)^ and λ, μ, *k*, and *l* are slope parameters tuned as per the model for mammalian cortical neuron**.

The activity of the Na^+^–K^+^ ATPase pump (*A*_pump_) is modeled by a combination of sigmoidal functions of the concentrations of ATP and K_o_ concentrations (Figure 3), assuming it to be independent of Na^+^ dynamics (Eq. 19). This is done so as to represent the significance of intracellular ATP and K_o_ in modulating the pump activity under pathological conditions (51). The formulation of Na^+^–K^+^ ATPase pump dynamics is similar to that implemented by Forrest et al. (52) with parameters adjusted for the current model.

(19) $$A_{\text{pump}}=\frac{1}{\left(1+e^{-\lambda (K_{o}-k)})(1+e^{-\mu (A-l)}\right)}$$

where λ, μ, *k*, and *l* are slope parameters, *A* represents the intracellular ATP concentration and is given by Eq. 20 with τ_A_ as the time constant for the ATP dynamics. The steady state value of intracellular neuronal ATP is constrained to 2 mM as per the experimental observations (53). The model can be adapted to different neuronal types by changing the slope parameters for ATP and K_o_ in the Eq. 19.

(20) $$\tau_{\text{A}}\frac{\text{d}A}{\text{dt}}=\varepsilon_{\text{p}}f-A_{\text{pump}}-\lambda_{\text{A}}A$$

where *f*, a quantity analogous to neural firing rate, is calculated according to Eq. 21. In Eq. 20, *f* controls ATP production *via* the proportionality constant ɛ_p_, the production signal calculated by integration of the membrane voltage (Eq. 21). Furthermore, the consumption term is given by the activity of the *A*_pump_ (Eq. 19). On the one hand, consumption being local (to the neuron) affects the membrane voltage on a faster time scale. On the other hand, the production signal influences the membrane voltage on a slower time scale, since it is an outcome of the activities within the glio-vascular system.

(21) $$\tau_{\text{p}}\frac{\text{d}f}{\text{dt}}=-\lambda_{\text{f}}f+H(V)$$

where λ_f_ is the damping coefficient, τ_p_ is the time constant of ATP production (signifying the delay arising through the processing of information in glio-vascular loop), and *H*(*V*) denotes a Heaviside function of membrane voltage, *V*.

The model shows different firing patterns by varying the *I*_ext_ along with production coefficient, ɛ_p_.

### 2.2 Model 2: Dynamic Threshold Neuro-Energy Model

**Figure 4:**
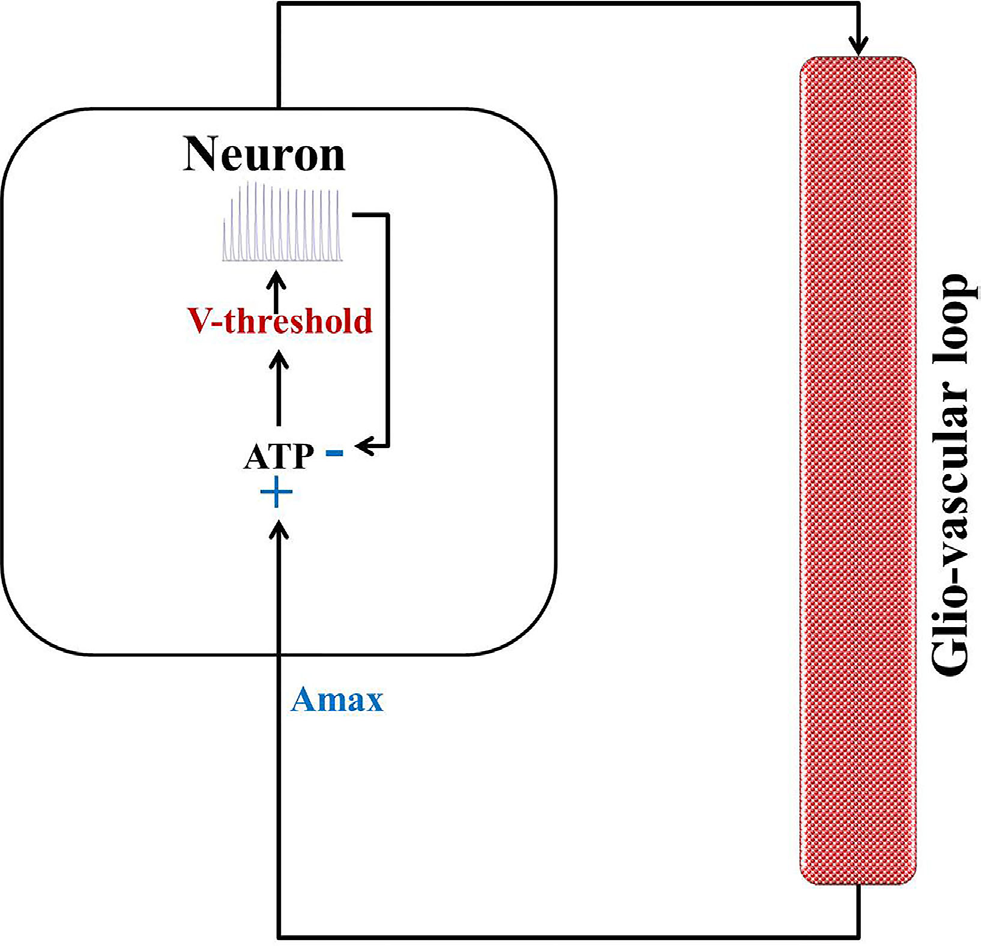
**Schematic representation of Model 2: dynamic threshold model**.

**Figure 5:**
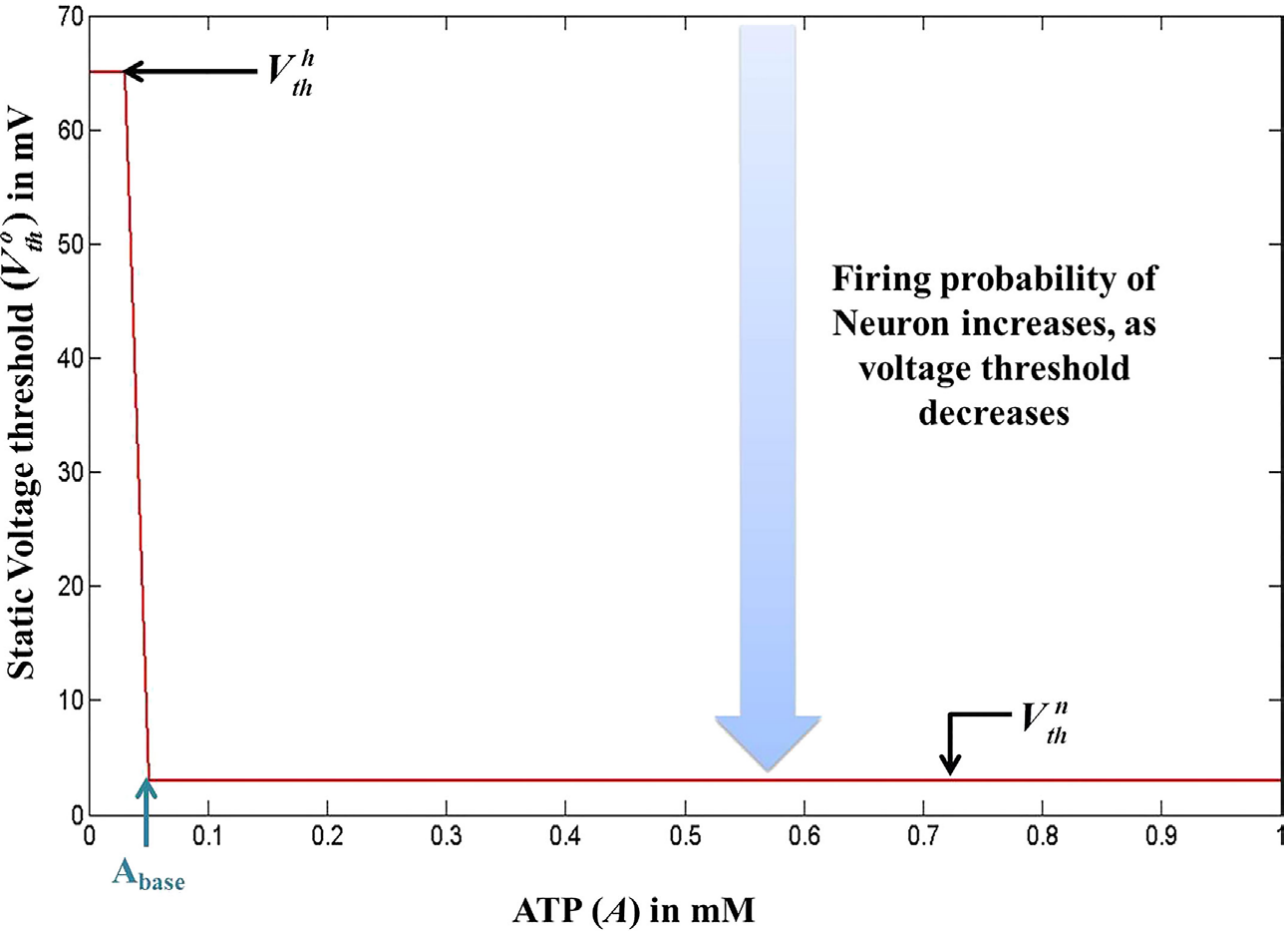
**Modeled voltage threshold function dependent on intracellular ATP concentrations (millimolar)**. The switch from $V_{\text{th}}^{\text{h}}$ to $V_{\text{th}}^{\text{n}}$ occurs at $A=A_{\text{base}}$.

In our quest to develop simple canonical models for NGV unit, we formulated the five-variable model described in Section 2.1, which depicts bursting behavior under energy-starved conditions. We then consider whether we could effectively replicate the behaviors of the previous model in a further simpler framework (Figure 4) and explore the plausible extent of abstraction, we now seek out a model that is simpler than that of Section 2.1, and reproduces the broad dynamic regimes and their transitions. The main intuitive idea behind the second model is that ATP controls the firing threshold of the neuron. The threshold is low for high ATP levels and increases under low ATP or energy-starved conditions (Figure 5). To this end, we construct the second model, which has three dynamic variables. This model comprises a quadratic integrate-and-fire neuron (Eq. 22), whose parameters are adjusted to obtain similar spike characteristics as that of the previous model (for a given *I*_ext_). The parameter and constants are listed in Table 2.

**Table 2:** **Table for values of different parameters and constants used in the formulation of Model 2**.

Parameters and constants	Value
*V*_reset_	−90 mV
*V*_peak_	30 mV
*V*_rest_	−100 mV
$V_{\text{th}}^{\text{high}}$	65 mV
$V_{\text{th}}^{\text{norm}}$	3 mV
*A*_dec_	0.07
*A*_base_	0.05

*All the parameter values are set to get the best alignment with the previous model in terms of behaviors for a range of parameters*.

(22) $$\tau_{\text{V}}\frac{\text{d}V}{\text{dt}}=a(V-V_{\text{th}})(V-V_{\text{rest}})+I_{\text{ext}}$$

where *V* is the membrane voltage, *V*_rest_ is the resting membrane potential, set to −100 mV (analogous to Model 1), and *V*_th_ is the dynamic voltage threshold that is dependent on the ATP inside the cell, given by Eqs 23 and 24.

The voltage is reset back to *V*_reset_ = −90 mV, when it reached the peak value, *V*_peak_ = 25 mV. The values of *V*_reset_ and *V*_peak_ were set to specific values in order to align the firing rates of Model 2 with that of Model 1.

(23) $$\tau_{\text{th}}\frac{\text{d}V_{\text{th}}}{\text{dt}}=-V_{\text{th}}+V_{\text{th}}^{\text{o}}(A)$$

where $V_{\text{th}}^{\text{o}}$ is a function of ATP, denoted by *A*.

(24) $$V_{\text{th}}^{\text{o}}(A)=V_{\text{th}}^{\text{n}}+(V_{\text{th}}^{\text{h}}-V_{\text{th}}^{\text{n}})H(A_{\text{base}}-A)$$

where $V_{\text{th}}^{\text{n}}$ is the basal of membrane voltage threshold, set to 3 mV for the present set of simulations, and *H*(*V*) is the Heaviside function of membrane voltage, *V*. Figure 5 shows the relationship between *A* and $V_{\text{th}}^{\text{o}}(A)$, wherein *V*_th_ takes a higher value $V_{\text{th}}^{\text{h}}$ (=65 mV) under low ATP conditions $\left(A<A_{\text{base}}\right)$, and a lower value, $V_{\text{th}}^{\text{n}}$ (basal threshold = 3 mV) for higher *A* (>*A*_base_). The ATP dynamics are modeled with a production term and a consumption term similar to that of Model 1. Here, the production term does not explicitly depend on the firing rate; instead, in the absence of firing activity, *A* approaches exponentially a maximal value of *A*_max_, assumed to be the physiological ceiling level of ATP (53).

(25) $$\tau_{\text{A}}\frac{\text{d}A}{\text{dt}}=\varepsilon_{\text{p}}(A_{\max}-A)-\delta (t^{\text{sp}}-t)A_{\text{dec}}$$

where τ_A_ is the time constant for ATP dynamics, *A*_dec_ is the step decrease in *A*, whenever the cell spikes, and finally, ɛ_p_ is the production coefficient analogous to the one used in the previous model. The present model is simplified and shows qualitatively the same range of behaviors as that of the previous biophysical model by varying ɛ_p_ along with *I*_ext_.

### 2.3 Neuro-Energy-Based Network Model

**Figure 6:**
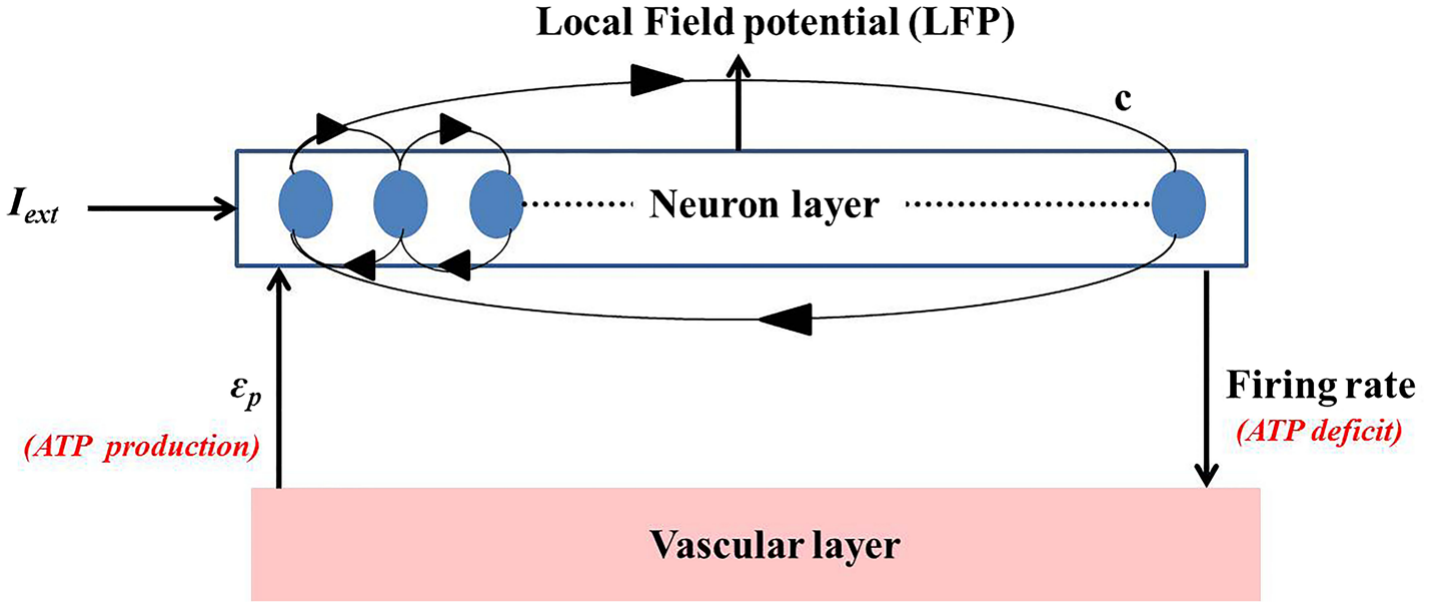
**Simulated network representation: the neuron layer comprises 1000 neurons with 15% as fast spiking inhibitory interneurons**. The activity of each neuron is coupled to the ATP dynamics. The connectivity between the neurons is random and sparse (*c* = 20%). ATP production rate for each neuron is governed by a production rate coefficient, ϵ_p_, proposed to be representing vascular input parameter. Neuronal firing consumes ATP, creating a deficit which results in increase in the production of ATP, representing the vascular feedback.

The neuro-energy unit as described in Section 2.2 is then implemented to construct a network model of 1000 such units in order to study the effect of the control parameter/s on the emergent properties of the network. The network configuration is similar to that implemented in the earlier studies (37, 54) and is illustrated in Figure 6. It comprises 85% excitatory neurons and 15% inhibitory neurons (55). Unlike Ching et al. (37), the inhibitory neurons also have the intracellular ATP dynamics associated with their activity, which in turn controls their firing threshold.

The basic neuronal model for both excitatory and inhibitory neuron is the quadratic integrate-and-fire neuron, with the major difference in the time constants (τ_excitatory_ = 2τ_inhibitory_). This ensures faster dynamics of the inhibitory neurons, representative of the fast spiking inhibitory neurons in the cortex. In addition, the neurons in the network are connected through synapses modeled as alpha function (Eqs 26 and 27), with the synaptic parameters (Table 3) corresponding to that of cortical pyramidal and fast spiking inhibitory neurons similar to Cunningham et al. (56).

**Table 3:** **Network parameters utilized for modeling the synaptic currents as adapted from Ching et al. (37)**.

	Pyrammidal	Fast-spiking interneurons
*g*_AMPA_	0.1	2
*g*_GABA_	0.64	1

(26) $$I_{\text{syn}}=g_{\text{i}}h(E_{\text{i}}-V)$$

(27) $$\tau_{\text{i}}\frac{\text{d}h}{\text{dt}}=-\lambda h+\delta (t-t^{\text{sp}})$$

where “*i*” represents the type of synapse (excitatory or inhibitory); *E*_AMPA_ and *E*_GABA_ are 0 and −80 mV, respectively. Synaptic currents, *I*_syn_, are calculated from the integral of the presynaptic spike history δ(*t* − *t*^sp^), which can be obtained by using auxiliary variable, *h*, similar to Mazzoni et al. (54) with the damping constant, λ, tuned as per this model. The values for *E* and *g* are taken from Ching et al. (37), while the value for τ was tuned in a way so that τ_ext_ = 0.5τ_in_ as the inhibitory synapse are at least twice as fast as excitatory synapses (37).

The corresponding *g*_i_ values for both types of synapses are given in Table 3. Furthermore, to maintain a conservative biological realism in the model, the connectivity was chosen to be random and sparse with a 0.2 probability of directed connection between any pair of neurons, similar to Mazzoni et al. (54). For the described set of parameters, LFPs are calculated as sum of synaptic currents, averaged over the neurons in the network and is given by the following equation:

(28) $$\text{LFP}=\frac{1}{N}\sum_{n=1}^{N}\sum_{i=1}^{C}I_{\text{syn}}^{i,n}$$

where *N* is the number of neurons in the network and each neuron receives input from *C* synapses (20% of the total number of synapses).

## 3 Simulation Results

Both the models show same set of behaviors for a similar range of parameters, *I*_ext_ and ɛ_p_. As ɛ_p_ corresponds to the vascular input to the neurons, we suggest that low ɛ_p_ would be representative of “energy deficient” condition in the neurons. Both the models show bursting (tonic/phasic) under low ɛ_p_ and moderate *I*_ext_ conditions, thereby suggesting metabolic basis of bursting. We demonstrate this effect in Model 1, wherein low ɛ_p_ conditions result in activations of K_ATP_ channels and thus show bursting at some moderate values of *I*_ext_.

### 3.1 Model 1: Simulation Results

**Figure 7:**
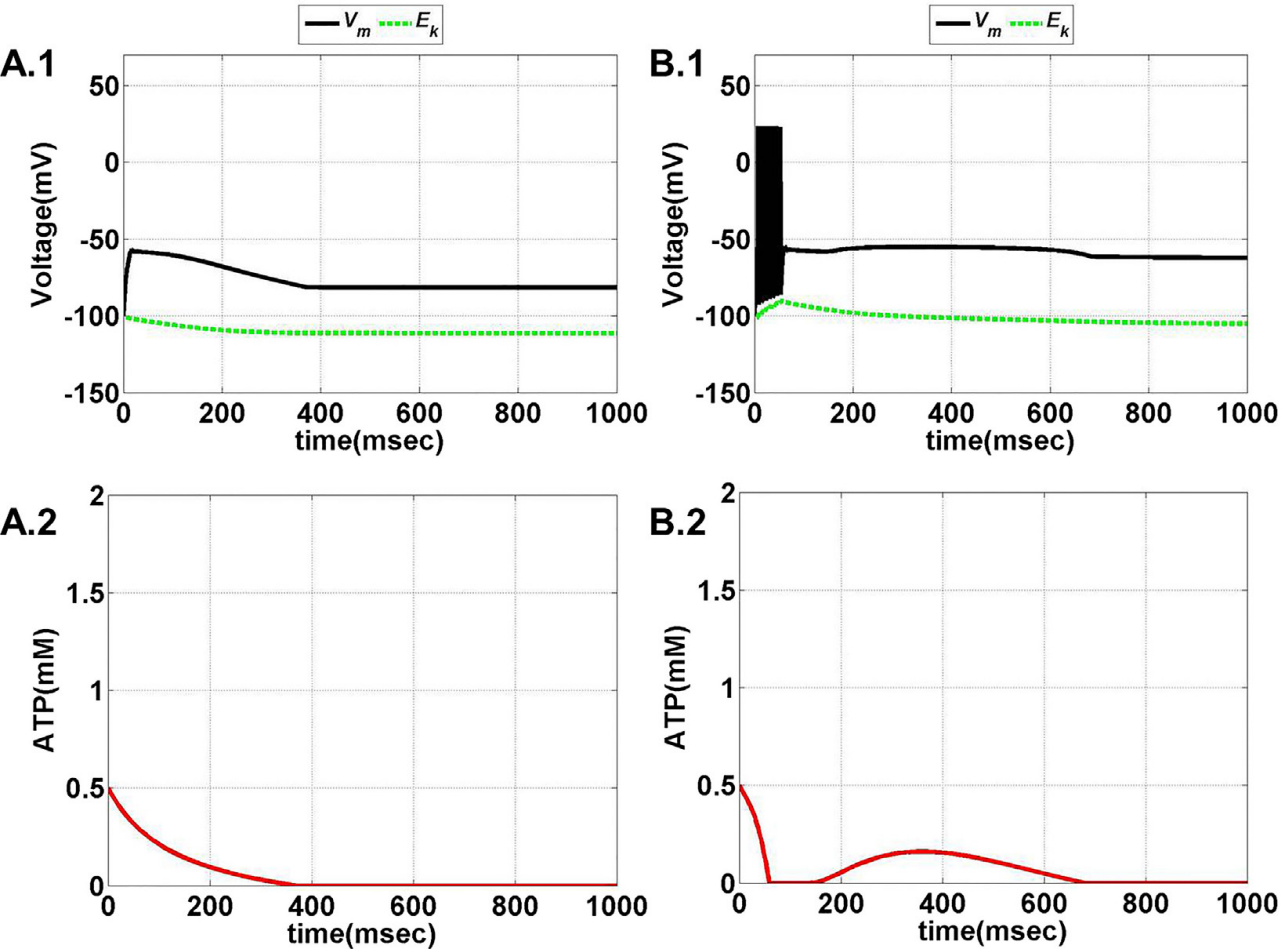
**Model 1: the membrane voltage dynamics (A.1,B.1) are shown as black traces**. The green trace represents the *E*_K_ (*Nernst potential for potassium*) dynamics for no firing and phasic bursting, respectively. The corresponding ATP dynamics as in **(A.2,B.2)** for respective regimes are illustrated with continuous red lines (simulated for 1 s). **(A)** No spiking at *I*_ext_ = 3 μA/cm^2^. Note that in this case, the value of ɛ_p_ affects the slope of the ATP dynamics, and not changing the overall behavior/regime. **(B)** Phasic bursting at ɛ_p_ = 0.8 and *I*_ext_ = 7 μA/cm^2^. As can be observed from the ATP dynamics in **(B.2)**, there is a slight rise in the [ATP], followed by complete silence, owing to low ɛ_p_ conditions.

**Figure 8:**
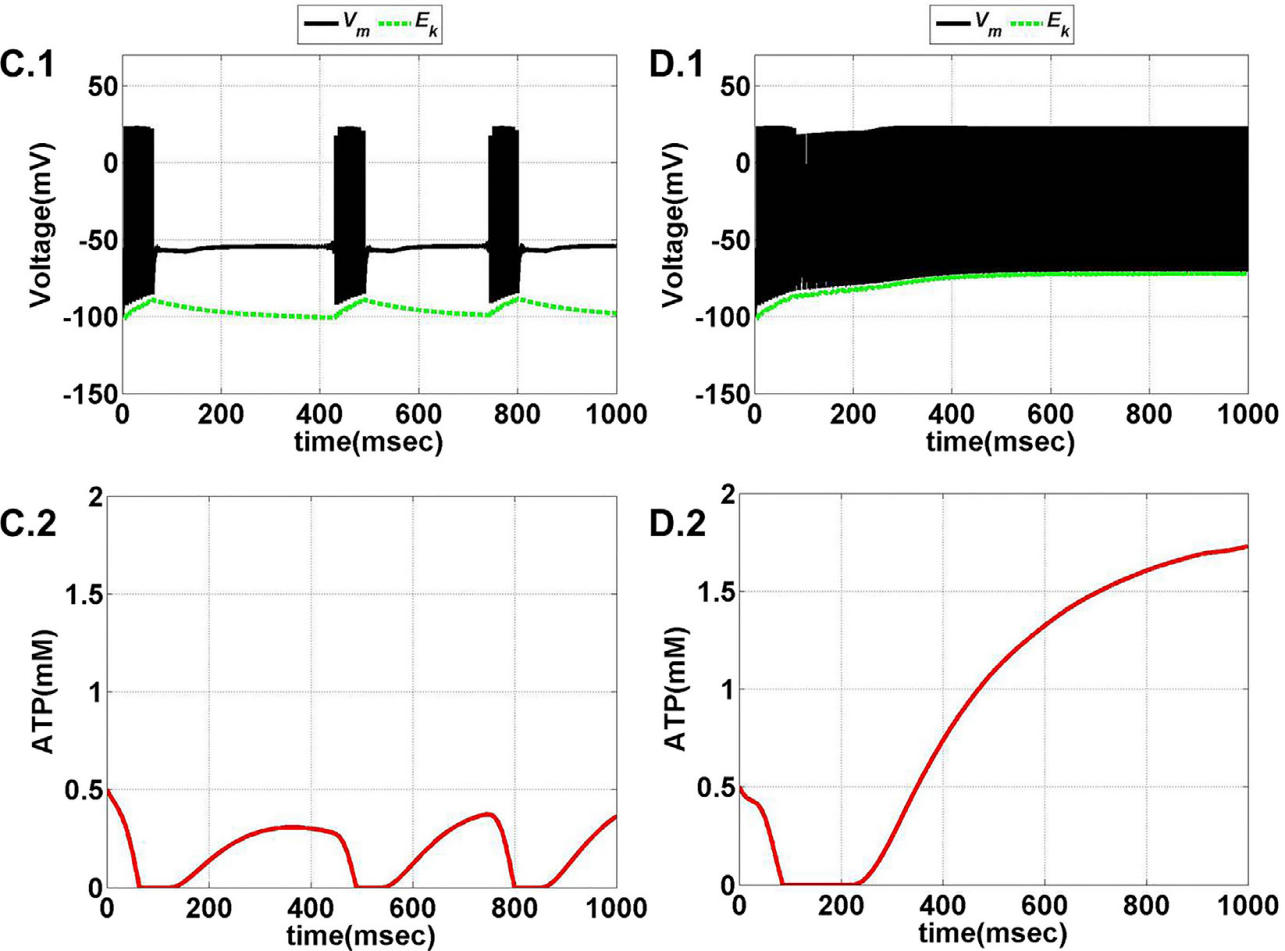
**Model 1: the membrane voltage dynamics (C.1,D.1) are shown as black traces**. The green trace represents the *E*_K_ (*Nernst potential for potassium*) dynamics for no firing and phasic bursting, respectively. The corresponding ATP dynamics **(C.2,D.2)** for respective regimes are illustrated with continuous red lines (simulated for 1 s). The green trace is that of the *E*_K_ dynamics, **(C)** tonic bursting at *I*_ext_ = 7 μA/cm^2^ and ɛ_p_ = 1. The ATP dynamics [as in **(C.2)**] clearly demonstrate the effect of fast-slow subsystems, i.e., the production is slower than the consumption. Consequently, the uphill is more stretched across time as compared to the downhill. **(D)** Continuous spiking at ɛ_p_ = 2 and *I*_ext_ = 7 μA/cm^2^. The initial dip in the ATP dynamics is characteristic of the fast local consumption; however, due to higher ɛ_p_, the demand for ATP is sufficiently matched with the supply and the intraneuronal ATP then reach to a homeostatic physiological value (=1.8 mM).

**Figure 9:**
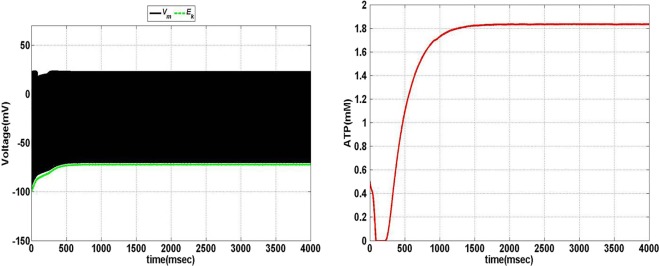
**Model 1: the membrane voltage dynamics (left) with corresponding ATP dynamics (right) for corresponding regimes at *I*_ext_ = 7 μA/cm^2^ and ɛ_p_ = 1.7 simulated for 4 s to show the stabilization of ATP over the time in case of continuous firing**.

Model 1, with its biophysical framework, shows four major behaviors by varying ɛ_p_ and *I*_ext_: *no firing*, *phasic bursting*, *tonic bursting*, and *continuous spiking*. In addition, it also shows *phasic spiking* (a single spike followed by a pause of fixed duration) for some values of ɛ_p_ and *I*_ext_ (traces not shown). Figures 7 and 8 illustrate the voltage dynamics and the corresponding ATP dynamics for the regimes mentioned above for Model 1. In addition to the voltage trace (black), the *E*_K_ dynamics is also depicted in the both the figures (in green). For any value of ɛ_p_ and subthreshold *I*_ext_, the neuron tends to be in a no-firing state as in Figure 7A.1. The value of ɛ_p_ becomes significant for suprathreshold *I*_ext_. For example, for *I*_ext_ >4 μA/cm^2^ and low values of ɛ_p_, the model shows (phasic or tonic) bursting (Figures 7B.1 and 8C.1), whereas for higher values of ɛ_p_, continuous spiking is observed (Figure 8D.1). The state as described in Figure 8D.1 is considered to be physiological state, wherein the cell is showing continuous activity for the given *I*_ext_ value. On the contrary, the states described in Figures 7B.1 and 8C.1 could be considered as pathological states, wherein the neuron is unable to fire for a long time/continuously due to lesser availability of energy/ATP (low ɛ_p_). The corresponding ATP dynamics (as in Figures 7A.2, B.2 and 8C.2, D.2) reveal fast and slow components in the sense that the ATP production is slower than its consumption. Particularly, in the case of continuous spiking, ATP increases slowly after the initial decrease, to stabilize at a value around 1.8 mM as shown in Figure 9. This state represents the physiological state as the demand of ATP is matched with the supply, maintaining a homeostatic [ATP] = 1.8 mM (53).

As mentioned before, in order to keep minimal variables in the model, we incorporated K_o_ in our model to emphasize the importance of K_o_ in pathological conditions. Interestingly, K_o_ was shown to be slowly accumulating during normal phase of firing (57), which is also demonstrated in the proposed model.

**Figure 10:**
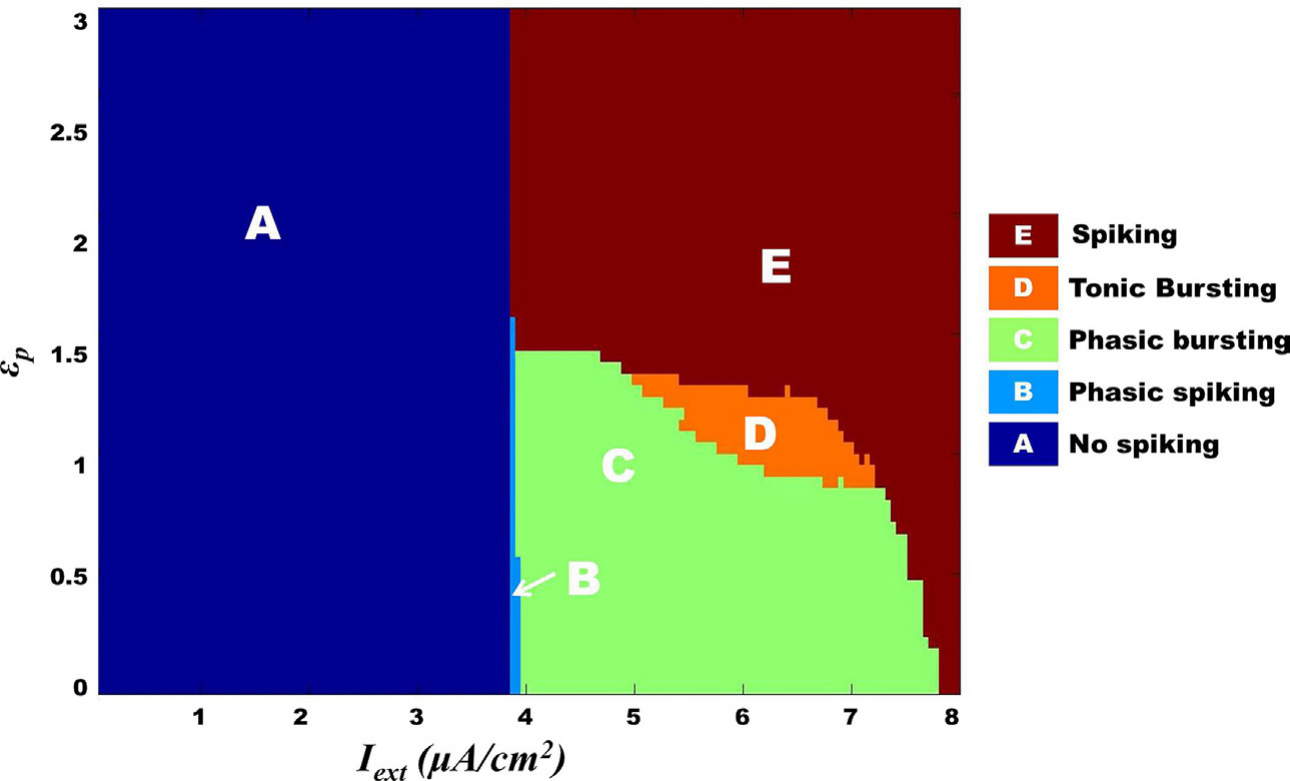
**Model 1: complete map of different regimes obtained by varying ɛ_p_ and *I*_ext_ simulated for 1 s**. The vertical boundary between regime A and the other regimes depicts the threshold value of *I*_ext_. Bursting (tonic/phasic) is observed only under low ɛ_p_ conditions and medial above threshold *I*_ext_. The direct switch from no spiking to continuous spiking (A–E) is considered to be physiological. Note that the phasic spiking regime (regime B) is dependent on the initial conditions of membrane potential set to −100 mV [similar to Cunningham et al. (56) and Ching et al. (37)].

We further show that the transition from one behavior to another is smooth with an intermediate regime, which shows a mixture of dynamics characteristic of the adjacent two regimes. Specifically, with respect to Model 1, such a transition zone was seen between the tonic bursting and continuous spiking regimes. Figure 10 illustrates the map for Model 1 showing the gross regimes obtained by varying the two control parameters: ɛ_p_ and *I*_ext_.

### 3.2 Model 2: Simulation Results

Model 2 is a lower dimensional model compared to Model 1. The decrease in ATP is modeled to be a step decrease and the production of ATP is similar to that modeled by Ching et al. (37). In addition, the production term is a slowly increasing term whose slope is governed by ɛ_p_, which we propose to be analogous to the glio-vascular input.

**Figure 11:**
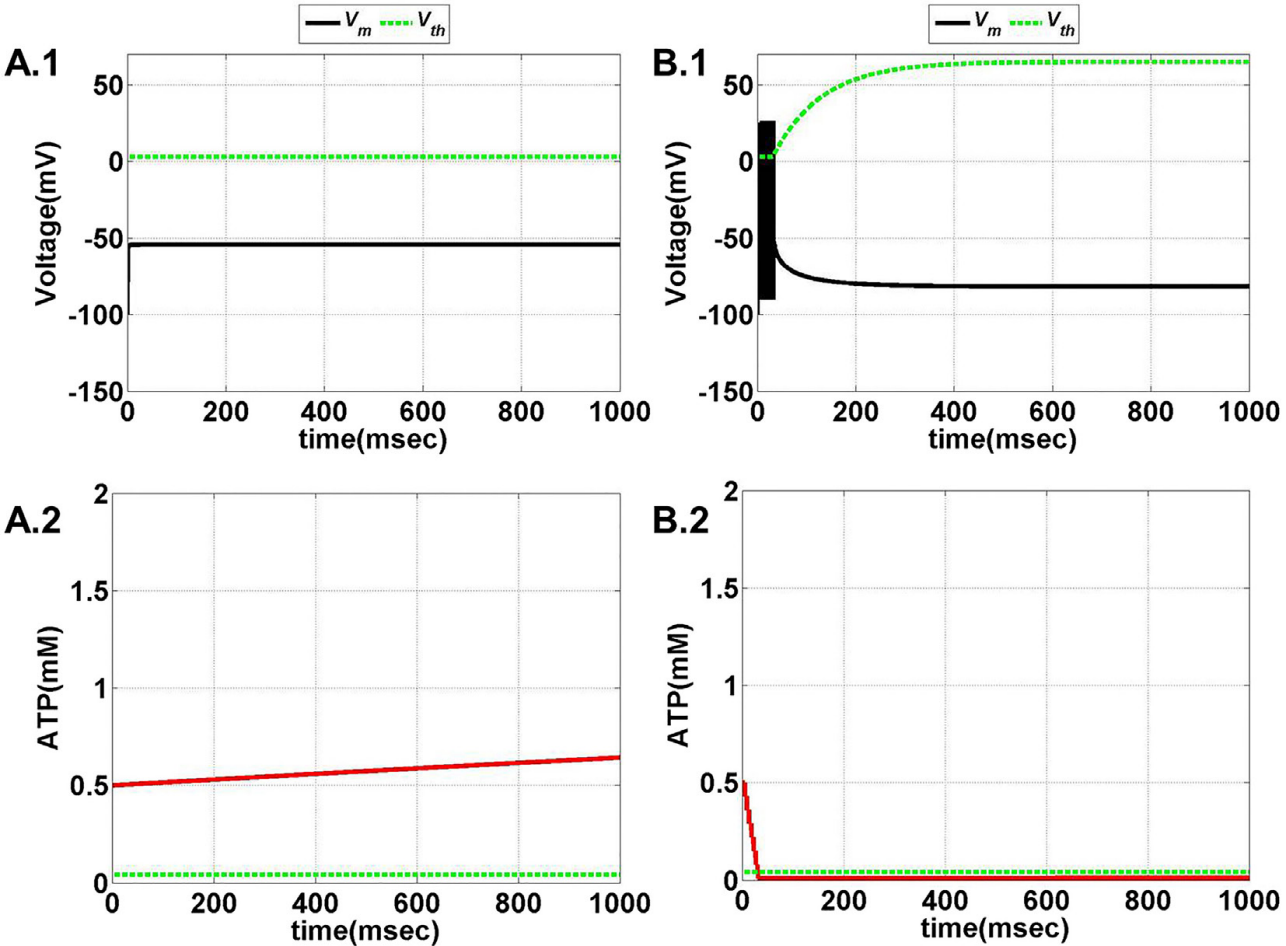
**Model 2: the membrane voltage dynamics (A.1,B.1) are illustrated in the black traces and the voltage threshold, *V*_th_ in the dashed green traces**. The corresponding ATP dynamics **(A.2,B.2)** for respective regimes are depicted by red traces with the dashed green trace representing the *A*_base_ (simulated for 1 s). The value of *A*_base_ is deterministic for the state of the neuron such that if *A* crosses *A*_base_, the threshold is raised to the higher value and neuron ceases to fire [**(B.1,B.2)**]. **(A)** No spiking at *I*_ext_ = 6 μA/cm^2^ for any value of ɛ_p_. **(B)** Phasic bursting at ɛ_p_ = 0.001 and *I*_ext_ = 7 μA/cm^2^. Here, the ɛ_p_ is very low to make the *A* cross the *A*_base_, and thus, the neuron tends to be in the phasic bursting mode.

**Figure 12:**
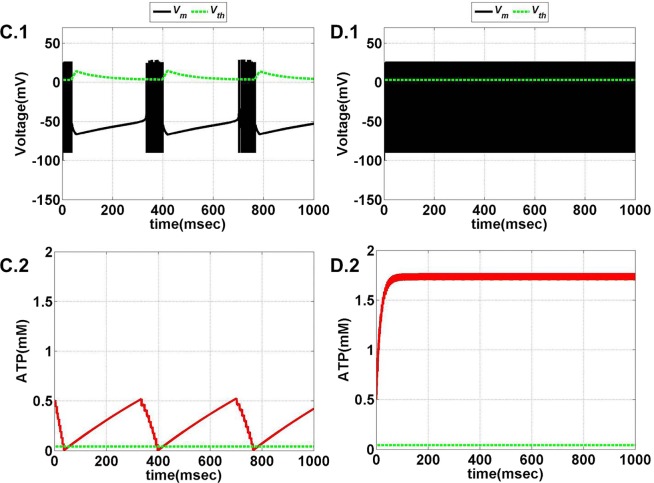
**Model 2: the membrane voltage dynamics (C.1,D.1) are illustrated in the black traces and the voltage threshold, *V*_th_ in the dashed green traces**. The corresponding ATP dynamics **(C.2,D.2)** for respective regimes are depicted by red traces with the dashed green trace representing the *A*_base_ (simulated for 1 s) and at *I*_ext_ = 7 μA/cm^2^. **(C)** Tonic bursting at ɛ_p_ = 1, note that here the decrease in ATP is a step decrease, and hence, the downhill of ATP is not smooth as compared to Model 1. **(D)** Continuous spiking at ɛ_p_ = 50, wherein the ATP stabilizes at the physiological value of 1.8 mM (ATP oscillates around the baseline of 1.8 mM).

The model is optimized such that variation of ɛ_p_ within the range of 0–5 displays all the behaviors observed in the previous model (Figures 11 and 12). Intuitively, the results suggest that there is a critical homeostatic balance between production and consumption of energy that has to be physiologically maintained by the coordinated activities of the three networks working in parallel: the neural, the glial, and the vascular network. The coordination is of great importance for generating complex spatiotemporal patterns of delivery of energy resources required for normal cerebral functioning.

**Figure 13:**
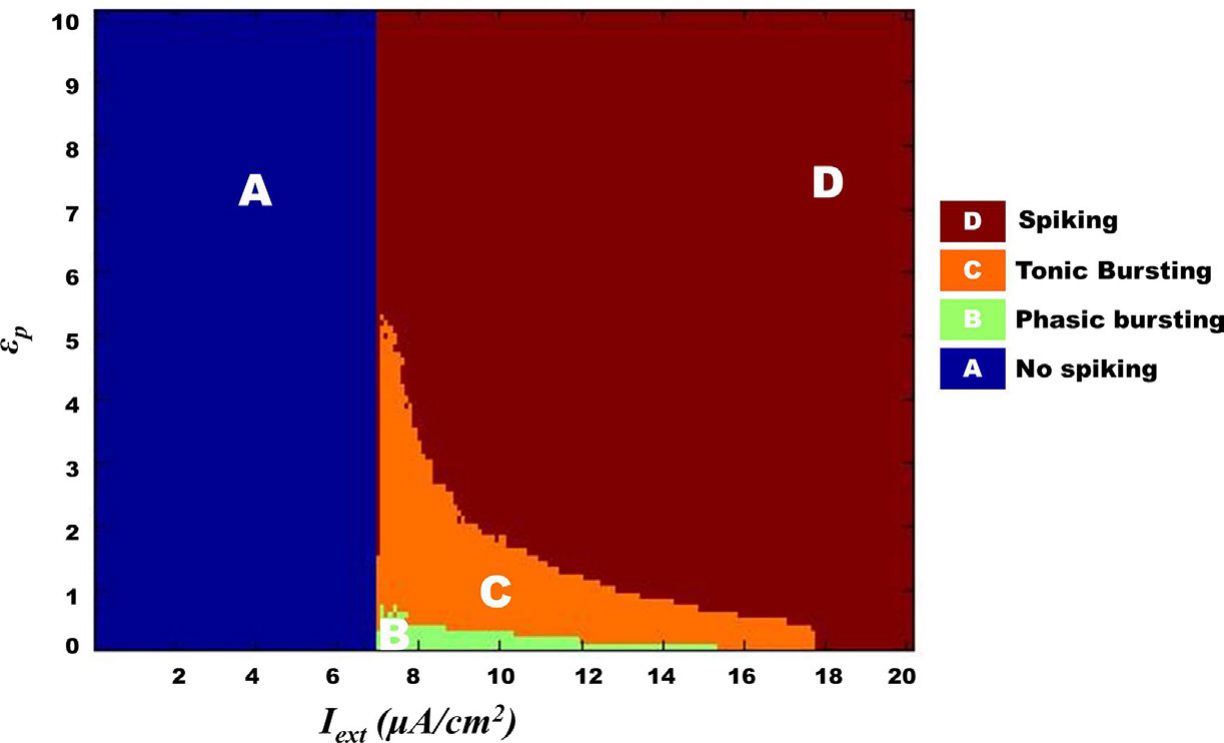
**Complete map for Model 2 regimes obtained by varying ɛ_p_ and *I*_ext_ simulated for 1 s**. The regimes transitions in the map are qualitatively similar to that observed in Model 1 (Figure 10) such that bursting (tonic/phasic) prevails under low ɛ_p_ and medial above threshold *I*_ext_.

Model 2 captures the essence of the previous model and faithfully reproduces the same range of behaviors, such as continuous spiking, phasic, and tonic bursting, by varying ɛ_p_ and *I*_ext_ as were observed in Model 1 (Figure 13). Figures 11 and 12 illustrate the various regimes and the corresponding ATP dynamics. The voltage threshold, *V*_th_ is also shown along with the membrane voltage traces (dashed green traces and black traces as in Figures 11A.1, B.1 and 12C.1, D.1, respectively). Correspondingly, the ATP traces are shown with the critical base ATP value (red traces and dashed green traces as in Figures 11A.2, B.2 and 12C.2, D.2). The characteristic transitions between the regimes (depending on the set of parameters) are analogous to Model 1. At subthreshold *I*_ext_, the neuron tends to be in a no-firing state (Figure 11A.1) and for suprathreshold *I*_ext_, the value of ɛ_p_ governs the firing pattern and physiological state of the neuron (Figures 11B.1 and 12C.1, D.1). However, the values of the parameters corresponding to different behaviors observed in the two models (Model 1 and Model 2) are not identical. One reason behind this could be the difference in the time scales of the two models, which were adjusted to align the two models. The values of the control parameters (*I*_ext_ and ɛ_p_) for which a range of behaviors is observed lack direct mapping to the physiological values, and thus, future experiments are required for validation.

Compared to Model 1, Model 2 represents an important step toward an abstract model. While Model 1 relates energy to neural firing *via* K_ATP_ channel, Model 2 relates the two through a more abstract parameter viz., “firing threshold.” Furthermore, Model 2 also has fewer dynamic variables than Model 1.

### 3.3 Model Comparisons

**Figure 14:**
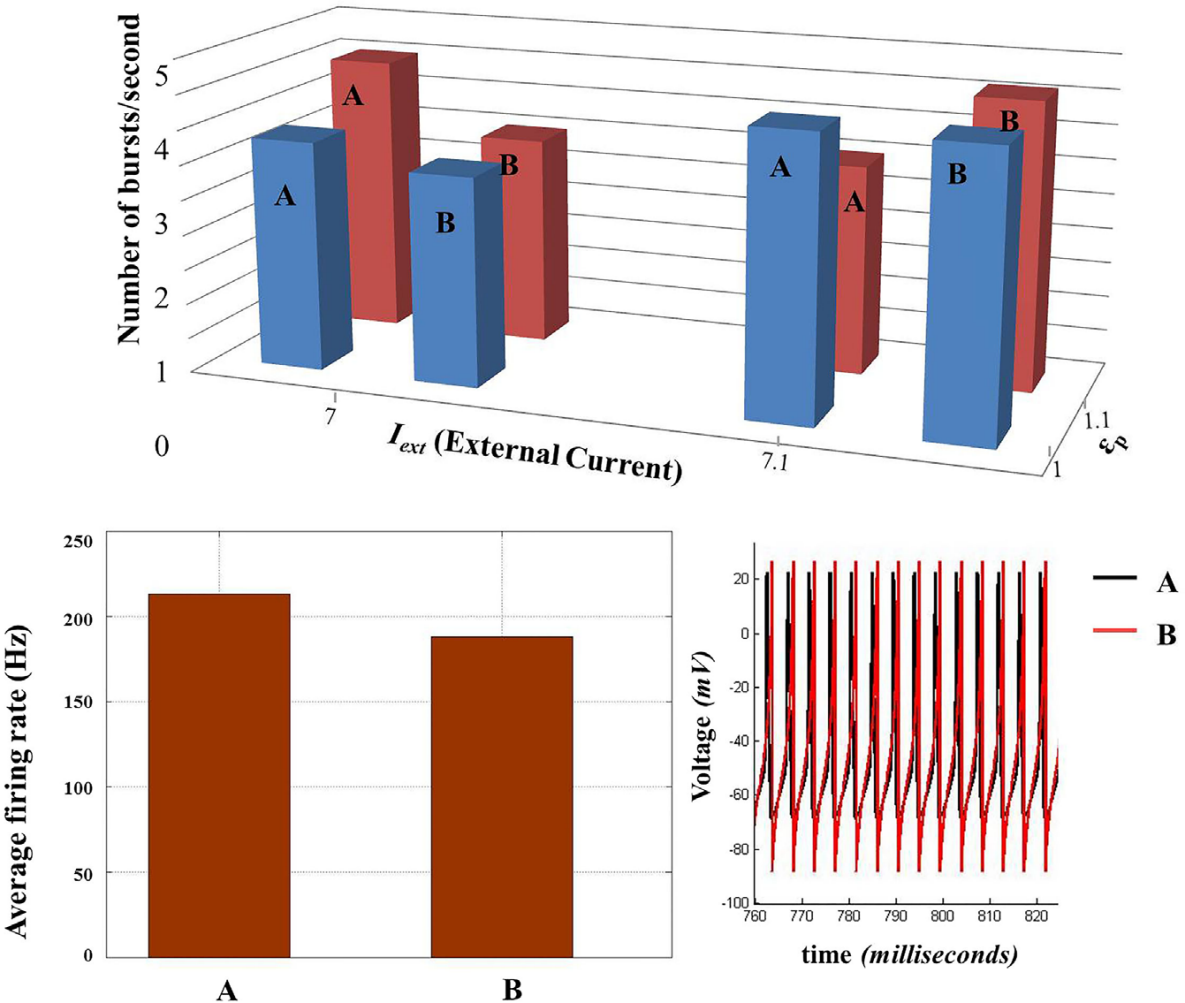
**Comparison of biophysical model (Model 1) (A) with the threshold model (Model 2) (B)**. (*Top*) Comparison of inter-burst frequency (number of burst per second) between the two models across two sets of control parameters (*I*_ext_ and ɛ_p_). (*Bottom left*) Comparison of average firing rate at *I*_ext_ = 8 and ɛ_p_ = 5. (*Bottom right*) The voltage traces of the two models compared. (*Since the average firing rate and inter-burst frequency are slightly different, the traces do not match completely throughout the time of simulation and for all the parameter configurations*.)

The parameters of Model 2 were tuned as per Model 1 so that the firing rates and burst rates for a given set of parameters match with good approximation. The firing rate comparison in shown in Figure 14 (top), while the individual spike dynamics is also shown in Figure 14 (bottom right). In addition, the inter-burst frequency is also comparable (Figure 14, bottom left) for a specific range of *I*_ext_. However, with the variation in $\varepsilon_{\text{p}}$, the match between the burst frequencies of the two models worsens (Figure 14: top red bar charts) due to the presence of aperiodic bursting in Model 2 (absent in Model 1). Consequently, the mean inter- and intra-burst frequencies are variable across the range of control parameters (both $\varepsilon_{\text{p}}$ and *I*_ext_). In general, the alignment between the models is consistent in terms of range of behaviors observed and their association with the control parameters.

### 3.4 Network Simulation Results

The production rate of ATP described by ɛ_p_ effectively changes the neural dynamics. Low ɛ_p_ is thought to represent the “metabolically compromised” network state and depicts that lower frequencies LFP dominate. This is similar to that observed under propofol-anesthetic conditions [associated with reduced metabolism (58, 59)], which is characterized by decreased LFP power (60–62). We further show that a broader spectrum of LFPs is observed at high ɛ_p_ conditions as opposed to the low ɛ_p_ conditions, wherein a sharper spectrum is observed.

The network output is analyzed in terms of LFP, population responses of excitatory and fast spiking inhibitory neurons, and the mean ATP corresponding to each population and network.

**Figure 15:**
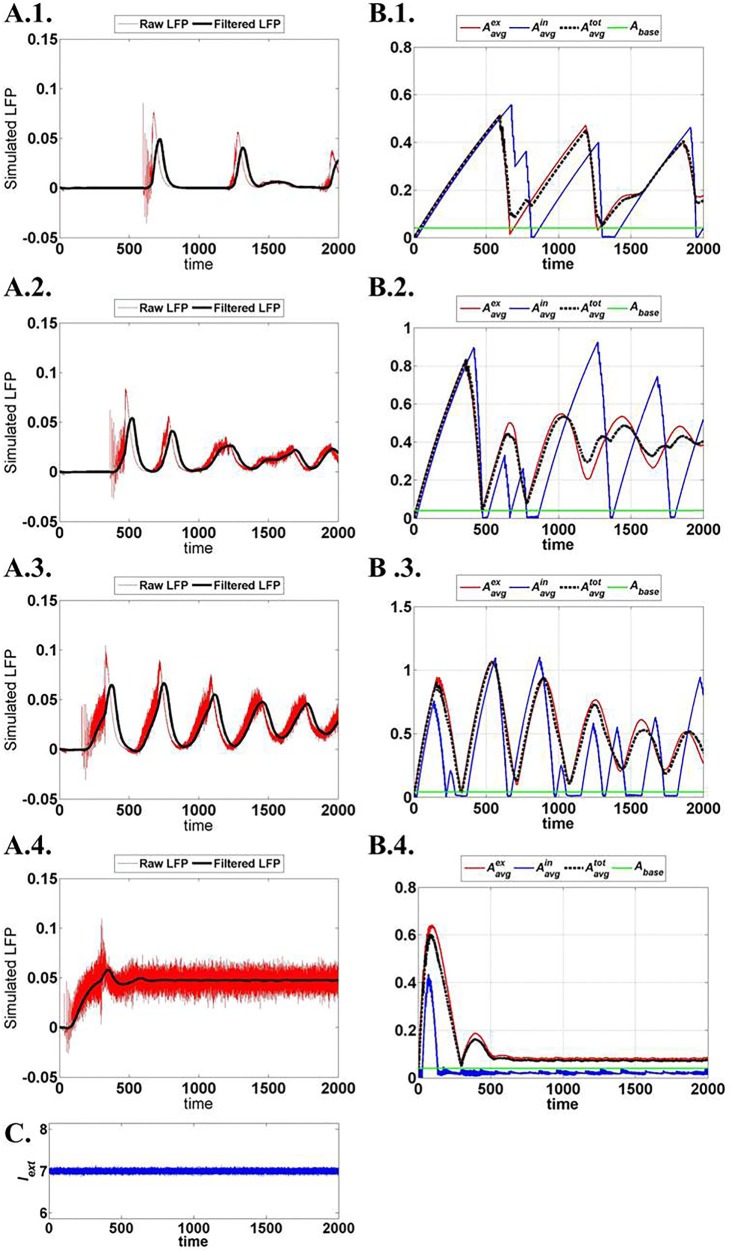
**Network output activity for increasing ɛ_p_: (A.1)–(A.4) raw and filtered LFP for ɛ_p_; 1, 3, 8, and 15 (top to bottom)**. **(B.1)**–**(B.4)** ATP dynamics averaged across neuron populations; inhibitory, excitatory, and complete layer for varying ɛ_p_. **(C)** External input current signal to the network with Gaussian noise.

**Figure 16:**
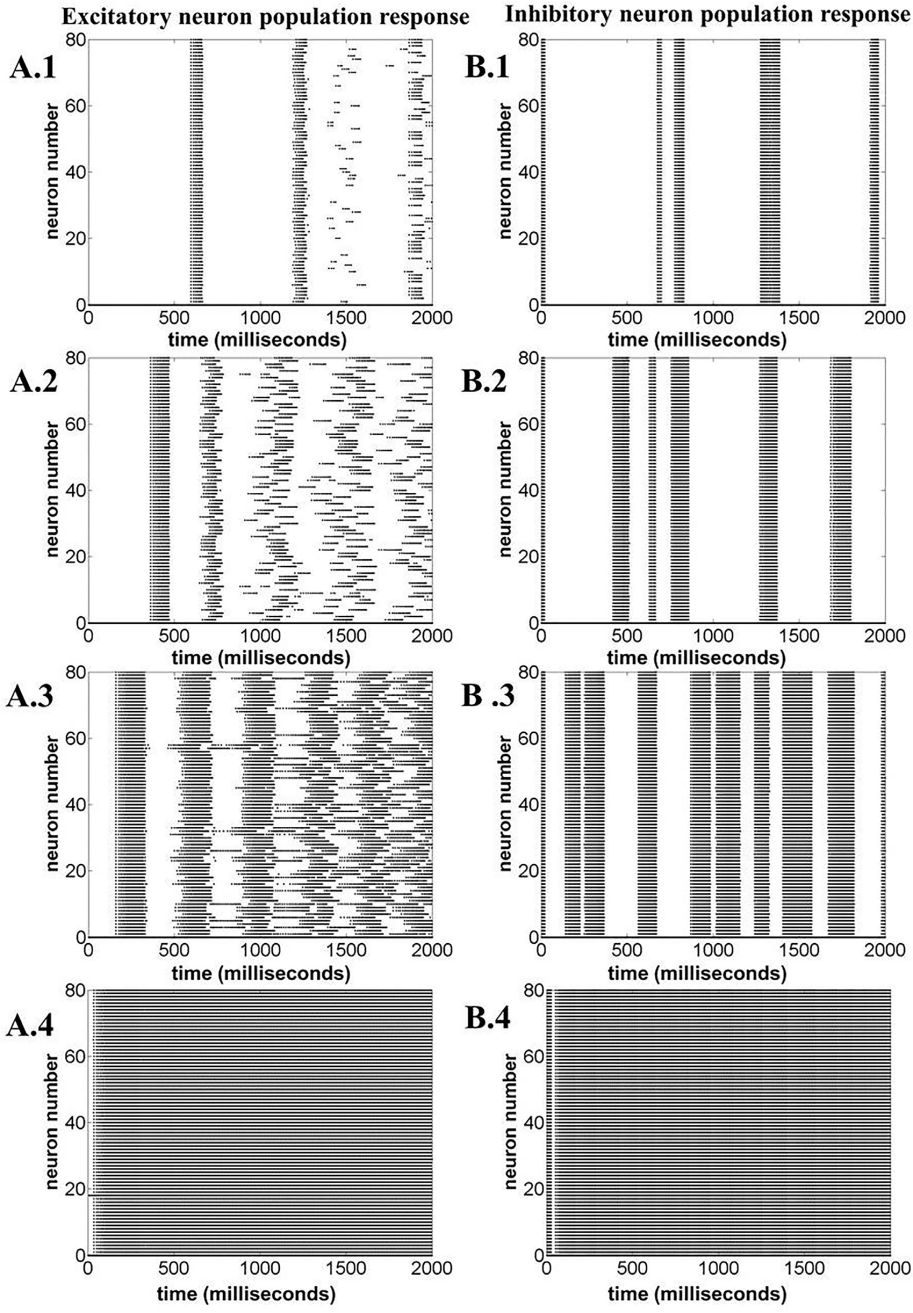
**Neuronal population response for varying ɛ_p_; 1, 3, 8, and 15 (top to bottom)**. **(A.1)**–**(A.4)** Excitatory neuron population response. **(B.1)**–**(B.4)** Inhibitory neuron population response. The figure illustrates that varying ɛ_p_ governs the synchronous firing among the excitatory neurons, making the network response more desynchronized at higher ɛ_p_.

Figure 15 illustrates the LFP patterns (Figure 15A.1–A.4) and mean ATP profiles for excitatory, inhibitory, and for the complete network, corresponding to various values of ɛ_p_. As can be observed, the baseline for mean ATP for the inhibitory neuron population is lower than that of the excitatory population. We believe that this happens as a result of relatively fast consumption of ATP by the inhibitory neurons as they display higher firing rates compared the excitatory/glutamatergic neurons. The fast consumption could be attributed to the synchronous population firing observed in case of inhibitory neurons as depicted in Figure 16, while the excitatory neuron population show desynchronous activation patterns. Higher ɛ_p_ shows continuous activation of both sets of neurons and is proposed to represent normal state (balanced excitation and inhibition).

**Figure 17:**
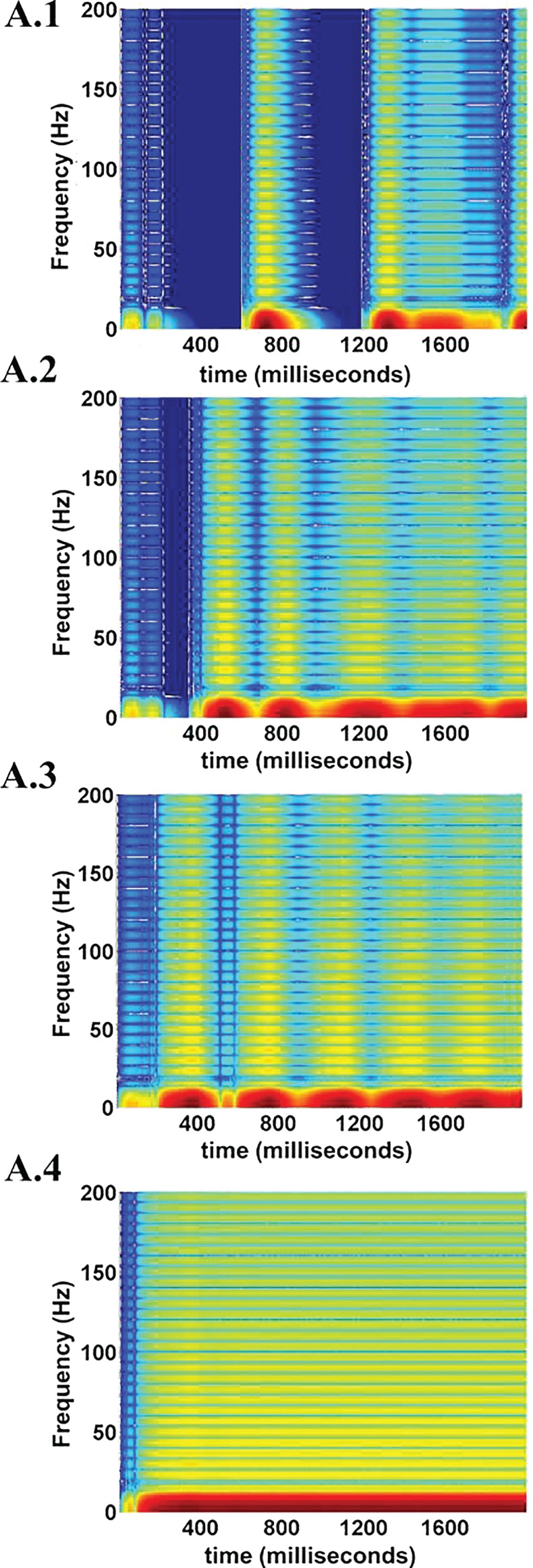
**LFP spectrograms for varying ɛ_p_; 1, 3, 8, and 15 (from A.1 to A.4)**. The effect of increasing ɛ_p_ is evident in terms of frequency range of LFPs. At low ɛ_p_ (=1) the LFP spectrum oscillates between high and low frequency (almost zero) components (A.1). Increasing ɛ_p_ results in a broader and less oscillatory LFP spectrum (A.2 and A.3). At very large values of ɛ_p_ rthe higher frequency components are uniformly seen throughout the simulation time period (A.4.).

The spectrogram for the calculated LFPs corresponding to different values of ɛ_p_ is shown in Figure 17, which shows the existence of high frequency activity with alternating periods of quiescence at low ɛ_p_ values. This is similar to the spectrum observed under deeper anesthetic conditions with isoflurane administration as described by Silva et al. (63), representing a metabolically compromised or low energy state of the brain.

## 4 Discussion

The present study proposes a minimal and computationally efficient model of the neurovascular system that could be used for simulating large scale networks. The first part of the study describes two low-dimensional single-unit neurovascular models depicting the importance of neuronal ATP production rate (representative of the vascular input) in modulating the neural firing patterns. As described earlier, the first model is biophysical and highlights the crucial link between the neuronal ATP and neural firing through the activity of the K_ATP_ channels. In order to simplify the first model further, based on the simple idea that “*ATP controls the firing threshold*,” we postulated the second model, a three-variable model. With a quadratic integrate-and-fire neuron model, the second neurovascular model parameters were adapted to mimic the spike characteristics observed in the first biophysical model. The second model effectively depicts the same set of behaviors observed in the first model as described in the previous sections. In the second part of the study, we then present a network model comprising the units described by the model equations of Section 2.2. With respect to the physiological correlates for the cortical neuronal networks, the simulated network is set to have 85% of the neurons as glutamatergic and 15% as GABAergic (55). The connectivity between the neuronal units is assumed to be sparse on the lines of earlier studies (64, 65). The simulation of the network shows that the LFP spectrum depends on the ATP production rate, ɛ_p_.

The described models specifically present the case of low ATP conditions (metabolic stress) in the neurons, wherein bursting (tonic/phasic) is observed at the single neuron level. Although there are studies showing burst suppression in electroencephalogram (EEG) as a result of reduced metabolic states, such as hypothermia, hypoxic–ischemic trauma (66, 67), there is no direct evidence showing bursting under such low ATP conditions at single neuronal level. However, there are experiments studying the activity of K_ATP_ channels under energy deficits or hypoxic conditions (68). Since K_ATP_ channel activations are associated with bursting both *in vitro* and *in vivo* (69, 70), this forms the crucial link between low ɛ_p_ and bursting. In the presented models, the neuronal system could show both tonic and phasic bursting. While most of the studies mentioned above demonstrate the occurrence of tonic bursting under reduced metabolic states, phasic bursting at the single neuron level is not studied well. However, there are studies showing that cervical interneurons transition from tonic bursting to phasic bursting under extreme hypoxic conditions (71). This highlights the key advantage of conducting computational modeling studies, wherein behaviors can be predicted and subsequently validated by experiments. Based on our simulation results, some of the testable predictions that suggest themselves are summarized as follows.

• The models described in the current study describe single neuronal behaviors. Hence, it would be pragmatic to first test the effect of “energy deficits” at the single neuron level experiments by designing a low energy conditions for the neurons followed by analysis of their activity. It would be also plausible to conduct such experiments on the slice cultures under low glucose and/or low oxygen conditions. It would be thus very interesting to see the change in the neuronal activity in these low energy conditions.
• The current study also suggests the existence of phasic bursting under extremely low energy conditions. Similar to the studies conducted by Sandhu et al. (71), experiments can be designed for cortical systems to investigate if the transition from tonic to phasic bursting occurs under extreme hypoxic conditions.
• An even more general experiment would be to observe the evolution of intracellular ATP during all the regimes described in the present study. Since there are few studies pertaining to the real time intracellular ATP measurements (69), it would be beneficial to measure the ATP levels along with the neuronal activity in the conditions suggested in the present study and look for oscillations in ATP occurring in synchrony with neural bursting.

At the network level models proposed in the present study, low-frequency large amplitude oscillations in LFP are observed with low ATP production rates. Studies depict a correlation between seizure activity or a spreading depression event and hypoxic–ischemic conditions for hippocampal neurons *in vitro* (72). We suggest that seizures or spreading depression observed in slice cultures could be linked to tonic bursting at single neuron level [similar suggestions have been made by Kager et al. (43)]. Furthermore, experiments by Sandhu and Gonzalez-Rothi (71) describe the impact of hypoxic condition on a subset of cervical interneurons and show a switch from tonic to phasic bursting with increasing level of hypoxia. The physiological evidence also associates the comatose conditions to the persistence of burst suppression activity in EEG (66). The neonatal seizures and EEG burst suppression also have been correlated to the manifestation of metabolically compromised state of the brain as a result of hypoventilation, hypoxic–ischemic encephalopathy, intracranial hemorrhage, or hypoglycemic conditions (73, 74). All these conditions are concomitant to reduced energy release by the vascular system or low cerebral blood flow rates, leading on to hyperexcitability of the cerebral cortex due to increased release of glutamate (75, 76). The excitability of the neurons is also known to be governed by the concentration of extracellular potassium, determined by the activity of the various potassium channels (49). Specific among the gamut of potassium channels, K_ATP_ is of specific interest as its activity is dependent on the energy state (intracellular [ATP]) of the neuron such that low energy inside the neuron is attributed to higher efflux of potassium from the neurons (77). Therefore, low intracellular energy would lead to hyper excitability as a result of either increased release of glutamate or higher potassium efflux from the neurons. These evidences reinforce our proposition of ATP controlling the neural firing threshold, thereby determining the excitability of the neurons. In the present study, the simulation of the network model depicts burst suppression, such as activity under low ATP production rate conditions, as demonstrated earlier.

It is thus evident that neuronal energy states are crucial in maintaining the physiological activity of the brain. We further speculate that these energy states must be considered in the background of the neurovascular coupling, as the neurons mostly depend on the surrounding glial cells and cerebral vasculature for energy supply (78). However, recent studies show the presence of glycogen metabolism in the neurons although it is thought to occur at meager levels under extreme cerebral hypoxia (79). Interestingly, the pathologies associated with the metabolically compromised brain states might possibly have an associated vascular dysfunction. Studies by Schwartz discuss the possibility of developing hemodynamic markers associated with the seizure onset in case of epilepsy (32). There exist clinical studies that also demonstrate occurrence of seizures in post-stroke cases (80), implicating vascular anomaly as one of the probable factors affecting the onset of epilepsy. Recent studies investigate the association between cerebral angiopathies and pathologies, such as Alzheimer’s disease, migraine, and epilepsy (81, 82). However, a clear causational relation between neurovascular pathophysiology and such disorders is not evident. For example, it is necessary to understand that whether the decrease in BOLD signal is associated with post-stroke seizure or it is an outcome of neuronal morbidity, as also discussed by Schwartz et al. (32). Thus, further studies are required to consolidate the notion of vascular dysfunction underlying cerebral pathologies. However, the “silent vascular infarcts” (also known as silent stroke) have been associated with dementia and neuropathies, such as vascular Parkinson’s disease (83–85). Such studies suggest that a primary vascular pathology may be a key factor precipitating neuronal dysfunction (82). The post-stroke studies depicting prolonged cognitive impairments (86) further corroborate the significance of the cerebral vasculature. All these studies highlight the notion that the vascular dysfunction could adversely affect the energy states of the neurons and therefore could predispose neurons to various pathologies.

The present study propounds a new perspective of energy coupled neuronal system, wherein the energy inside the neuron varies dynamically, depending on the neural activity and the local vascular influence. We further propose that this vascular influence boils down to the production rate of ATP inside the neurons, which is controlled by the production coefficient, ɛ_p_, in the models. This study presents low-dimensional models for neurovascular coupling, wherein glio-vascular system is lumped into an energy reservoir and the coupling is represented by ɛ_p_. Such models can be implemented to simulate network models to study the effect of neurovascular coupling on the network activity as the one described in the present study.

Another plausible direction of the study could be to introduce explicit vascular dynamics controlling ɛ_p_ associated with the neurons. The vascular component in such a neurovascular network model may be designed on the lines of the existing vascular network models (87–91). Significant among these models is the vascular anatomic network (VAN) model proposed by Boas et al. (90) as it is relatively simple and is able to qualitatively predict the BOLD response characteristics. The model proposed in the present study provides an optimal substrate for developing comprehensive neurovascular models, wherein the evolution of neural dynamics in terms of EEG/LFP can be studied along with corresponding vascular responses in terms of BOLD signals. The firing rate signal calculated from the neural network would govern the vasodilatory signal that is fed to the VAN. The cerebral metabolic rate of oxygen (CMRO_2_) calculated from the model set parameters can then be employed to control the ATP production rates, ɛ_p_ of the neurons, completing the picture of neurovascular coupling. Thus, VAN coupled effectively to the model demonstrated in the present study would provide the basis for understanding and simulating the data obtained from multimodal systems, such as EEG coupled to functional magnetic resonance imaging (EEG–fMRI) and EEG coupled to functional near infrared spectroscopy (EEG–fNIRS).

In particular, this genre of neurovascular models will prove to be a vital brain mapping tool and could be implemented to understand the functional mechanisms of post-stroke recovery. As described earlier, there are clinical studies depicting the existence of post-stroke seizures and spreading depression, such as events which affect the post-stroke rehabilitation (92–94). Studies have shown the association between the early onset seizures post-stroke with factors, such as metabolic dysfunction, glutamate excitotoxicity, hypoxia, and global hypoperfusion (80, 95, 96). These events are associated with cellular processes including ATP deprivation, mitochondrial dysfunction, reactive oxygen (RO), and nitrogen species production that further result in irreversible neuronal damage causing larger cortical lesions and impairing the recovery process (80, 95, 97–99). With the vascular component coupled to the network model presented in this study, the vascular dynamics resulting in low ɛ_p_ can be investigated quantitatively (as the low ɛ_p_ conditions underpin seizure-like activity as observed in our model simulations). This would further lead to effective monitoring of stroke rehabilitation, wherein the epileptic seizure-like event could be anticipated. Moreover, a clear association could be established between specific hemodynamic patterns and seizure-like events. Furthermore, with a neurovascular model, the downstream effects of non-invasive brain stimulation (NIBS), an add-on stroke rehabilitation procedure to promote motor function recovery, could be simulated. Major efforts are being undertaken in devising optimal stimulation parameters for NIBS and to make it suitable to specific stroke patient groups (100, 101). Therefore, there is an immediate need to have a computational simulation platform, wherein the patient-specific stroke conditions can be simulated and the rehabilitation procedures can be calibrated by deriving the appropriate and patient-specific stimulation parameter set. The present study hence forms the base for developing such simulation platforms for stroke rehabilitation procedures.

In general, this line of study would help in deciphering the vascular basis of the all the brain functions and would also lead to vascular-based frontiers for diagnosis and treatment of brain pathologies. Another interesting line of extension pertaining to the present study could be to further reduce the current low-dimensional models to two-variable models for neurovascular coupling, which would be amendable to phase plane analysis. This is important as it would provide an insight into the mathematical basis of complex neuronal behaviors (such as bursting) and also how vascular system/energy parameters affect such behaviors.

## Author Contributions

KC and VC: computational model development, analysis, and manuscript preparation.

## Conflict of Interest Statement

The authors declare that the research was conducted in the absence of any commercial or financial relationships that could be construed as a potential conflict of interest.
